# Preliminary study of wing interference patterns (WIPs) in some species of soft scale (Hemiptera, Sternorrhyncha, Coccoidea, Coccidae)

**DOI:** 10.3897/zookeys.319.4219

**Published:** 2013-07-30

**Authors:** Ewa Simon

**Affiliations:** 1 Department of Zoology, University of Silesia, Bankowa 9, 40-007 Katowice, Poland

**Keywords:** Scale insects, Coccidae, wings, males, WIP, interference colour patterns

## Abstract

The fore wings of scale insect males possess reduced venation compared with other insects and the homologies of remaining veins are controversial. The hind wings are reduced to hamulohalterae. When adult males are prepared using the standard methods adopted to females and nymphs, i.e. using KOH to clear the specimens, the wings become damaged or deformed, an so these structures are not usually described or illustrated in publications. The present study used dry males belonging to seven species of the family Coccidae to check the presence of stable, structural colour patterns of the wings. The visibility of the wing interference patterns (WIP), discovered in Hymenoptera and Diptera species, is affected by the way the insects display their wings against various backgrounds with different light properties. This frequently occurring taxonomically specific pattern is caused by uneven membrane thickness and hair placement, and also is stabilized and reinforced by microstructures of the wing, such as membrane corrugations and the shape of cells. The semitransparent scale insect’s fore wings possess WIPs and they are taxonomically specific. It is very possible that WIPs will be an additional and helpful trait for the identification of species, which in case of males specimens is quite difficult, because recent coccidology is based almost entirely on the morphology of adult females.

## Introduction

The superfamily Coccoidea or scale insects contains 7500 species of plant feeding hempiterans, comprising 48 families (according to ScaleNet data base). Many of them are economically important pest to agriculture, horticulture and forestry (Gullan and Cook 2007).

Within this superfamily, there is a very marked dimorphism between the adult male and female, both in their morphology and life histories, such that it is impossible to identify the male and female of the same species (or even family) using the same combination of characters (Koteja 1996).

Adult females are sack-like, with the head, thorax and abdomen fused together. They are all wingless and many have reduced legs and antennae but the mouth parts are usually well developed. They can be quite long lived, surviving for several months on their host plants. The adult males are delicate, ephemeral insects without mouth parts, and so live as adults for only a few days. They are usually alate, although characterized by diptery, and resemble small delicate flies (Gullan and Martin 2009).

Species of Coccoidea are almost always described based on female structure, whilst most males are so poorly known that they are mostly unidentifiable to species (Hodgson and Foldi 2006). For example, within the entire Coccidae family, the adult males of only about 90 species have been described adequately to date as they are rarely collected and seldom studied. In contrast, the adult females of approximately 1200 species have been well described in just the family Coccidae (Hodgson and Henderson 2004).

As was mentioned above, only male Coccoidea possess wings. These structures have a very simplified venation in the fore wings whilst the hind wings are reduced to hamulohalterae. Because of this simplification and because the wings have been lost from all females, it was considered that the wings were featureless and therefore their morphology has been neglected (Szelegiewicz 1971).

**Figures 1–5.**
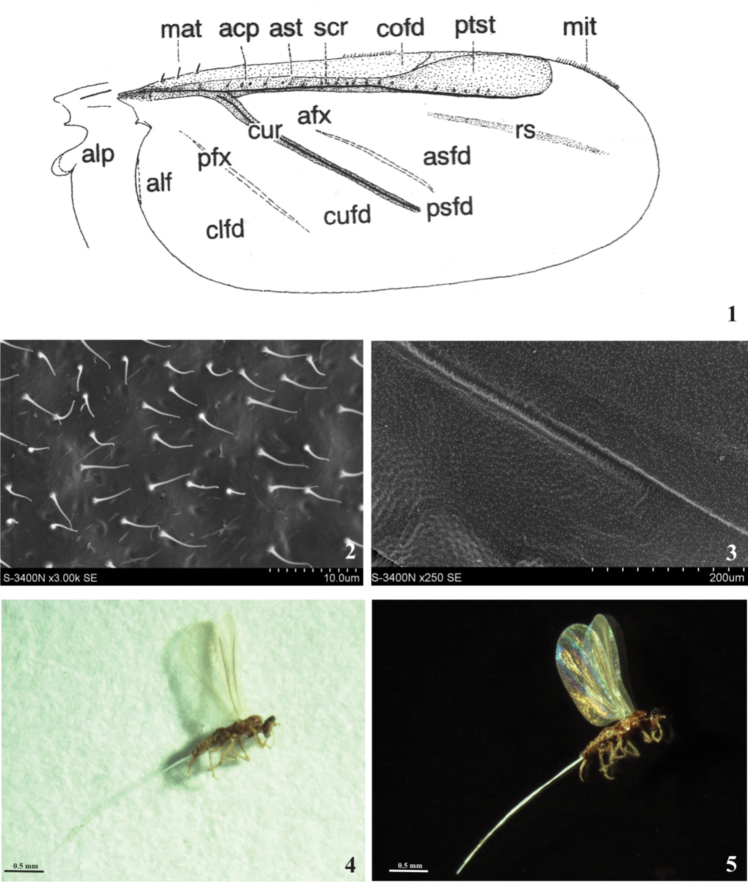
**1** General scheme of the fore wing (after Koteja 2008) acp-alar cupolae, afx-anterior flexing patch, alf-alar fold, alp-alar lobe, asfd-anterior subcostal field, ast-alar setae, clfd-claval (anal) field, cofd-costal field or thickening, cufd-cubital field, cur-cubital ridge, mat-macrotrichia, mit-microtrichia, pfx-posterior flexing patch, psfd-posterior subcostal field, ptst-pterostigma, rs-“radial sector”, scr-subcostal ridge **2, 3** *Pulvinaria vitis* (Linnaeus): scanning electron microphotographs of the wing, showing its microsculpture **4, 5** Male of *Pulvinaria vitis* (Linnaeus) on white background, with invisible WIPs, and 2 on a black background, showing WIPs.

The males are polymorphic with respect to the wings, i.e. winged, brachypterous, and wingless forms have been accepted by natural selection. During further evolution and adaptation to different ecological conditions the polymorphism has been retained or one morph has been preferred. Wing and wingless form may occur in different groups or within the same taxon, sometimes even in the same species (Kosztarab and Kozar 1988, Koteja 1985, 1996). The terminology connected with the wing morphology used here is that of Koteja (1996, 2008) and the general scheme of the fore wing presented on Fig. 1 comes from the Koteja’s work (2008).

The anterior wings are folded flat and overlap along the abdomen in resting position which facilitates the moving among soil particles, plant parts, etc. Shape of the wing is usually oval, but also with parallel anterior and posterior margins, with distal part wider than proximal one or reversely; with broad or narrow base, with rounded or acute apex.

For reception hamuli serves the narrow anal fold or projecting alar lobe with a pocket. On the wing there are two ridges: subcostal ridge which runs along costal margin from wing base toward wing apex; cubital ridge which originates from the former at about 1/5 wing length and runs obliquely to posterior wing margin. On the membrane of the wing might be present flexing patches (light lines) anterior – between subcostal ridge and cubital ridge and posterior – between cubital ridge and posterior wing margin. A slightly sclerotized oblique patch, which runs posterior to subcostal ridge is called radial sector. The ridges and flexing membranes divide the wing into fields, which are illustrated on Fig. 1. Depending on the development of ridges and flexing patches the fields assume various shapes, size, and may join or disappear. Pterostigma is present in some Archaeococcids and is hypodermal club-shaped thickening in front, or behind, subcostal ridge, not bordered with any veins (Koteja 2008). In all Neococcids and few groups of Archaeococcids the surface of the wing is covered with small hairlike microtrichia. Along the anterior margin of the subcostal ridge might be present a row or cluster of cupolae and alar setae might be interspersed among them or occur at the wing base (Koteja 1996).

Compared with most other structures, the wings of scale insects are best preserved dried or as fossils and have thus been found to be important in paleontological studies. The wings of recent specimens, prepared using standard methods adopted to females and nymphs (i.e. using KOH solution), become deformed or damaged, and usually are not described or drawn in publications (Koteja 2008). The present study looks at a new character present on scale insects wings, which has never been examined before in coccidology - the interference wing patterns (WIPs) of soft scales (Coccidae). Figs 4 and 5 illustrate the changes in the visual appearance of the wings (of *Pulvinaria vitis*) when the background is switched from white to black. WIPs occur on transparent wings with a very thin membrane, i.e. mainly in small insects. The colour patterns appear when wings are viewed against a dark background and show the uneven thickness of the wing membrane (Shevtsova 2012). The thickness of the composite chitinous membrane varies with the topography of the wing and the areas of different thickness reflect different interference colours that together produce a specific colour pattern, the WIP. Taxon specific colour patterns depend also on pigmentation, venation, and hair placement. The optically refracted pattern is also stabilized by membrane corrugations and spherical cell structure (Shevtsova et al. 2011). Observations of WIPs in many groups of Hymenoptera and Diptera suggest that species identification is enhanced if WIPs are added to the taxonomic characters (Shevtsova and Hansson 2011, Hansson 2011, Hansson and Shevtsova 2012).

## Material and methods

WIPs of seven species of the family Coccidae belonging to six genera were studied: *Eriopeltis lichtensteini* Signoret, *Eulecanium tiliae* (Linnaeus), *Luzulaspis frontalis* Green, *Luzulaspis nemorosa* Koteja, *Parthenolecanium corni* (Bouche), *Pulvinaria vitis* (Linnaeus) and *Sphaerolecanium prunastri* Boyer de Fonscolombe. This material comes from Koteja’s collection of scale insects deposited in Department of Zoology (University of Silesia, Katowice, Poland), and from author’s collection (only puparia with accompanied females were collected) (Table 1).

**Table 1.** The collection data of the studied material (KC – Koteja’s collection, AC – author’s collection).

**species**	**number of specimens studied**	**collection**	**date of collection**	**place of collection**	**plant**	**geographical coordinates**
*Eriopeltis lichtensteini* Signoret, 1877	25	KC	28.7.1967	Makowska Gora near Sucha, Poland	*Agrostis vulgaris*	
			20.09.1968	-	-	
			31.07.1969	Mikoszewo, Poland	different grasses	
			05.08.1969	Mikoszewo, Poland	*Calamagrostis epigejos*	
*Eulecanium tiliae* (Linnaeus, 1758)	5	AC	02.05.2012	Ruda Slaska, Poland	*Acer platanoides*	50°15'24.12"N, 18°54'5.64"E
			16.05.2012	Niegowonice, Poland	*Tilia cordata*	50°23'55.69"N, 19°26'8.92"E
*Luzulaspis frontalis* Green, 1928	13	KC	06.1962	Wolski Forest Cracov, Poland	*Carex brizoides*	
*Luzulaspis nemorosa* Koteja, 1966	15	KC	21.08.1967	Ojcow Cracov, Poland	*Luzula nemorosa*	
*Parthenolecanium corni* (Bouché, 1844)	6	AC	30.04.2007	Ruda Slaska, Poland	*Tilia cordata*	50°16' 5.24"N, 18°53'8.64"E
			07.05.2012	Ruda Slaska, Poland	*Ulmus laevis*	50°15'23.85"N, 18°54'6.42"E
*Pulvinaria vitis* (Linnaeus, 1758)	31	AC, KC	29.08.1987	Ruda Rozaniecka Roztocze , Poland	*Salix* sp.	
			26.08.1987	Ruda Rozaniecka Roztocze, Poland	*Betula verrucosa*	
			21.08.2011	Katowice, Poland	*Alnus glutinosa*	50°14'3.77"N, 19°0'55.71"E
*Sphaerolecanium prunastri* (Boyer de Fonscolombe, 1834)	6	AC	02.06.2012	Kuznia Raciborska, Poland	*Prunus spinosa*	50°12'9.02"N, 18°19'56.48"E
			16.05.2012	Niegowonice, Poland	*Prunus spinosa*	50°23'54.86"N, 19°26'15.78"E

The method used for preparation of the wings was standardized, as suggested by Shevtsova and Hansson (2011), i.e. dry wings were horizontally arranged (with the magnification 2 or 3×) and, flattened between a glass slide and glass cover. The underside of the glass slide was stained with black ink to make a pitch black background.

Photos of the wings were taken with a Nikon DN 100 camera unit on a Nikon stereomicroscopes SM2 1500.

Photos from scanning microscope were taken from a Hitachi S-3400N microscope and made in the Department of Materials Science of the Silesia University of Technology.

The brightness was individually adjusted in COREL DRAW X, subsequent editing included cleaning and cropping the photo. The studied wings were put between two cover glasses (glued together by small drops of transparent nail polish) and deposited with the original specimen.

The wings were also observed on whole specimens. In order to ensure that the colour patterns are stable, the wings on the unmounted specimens were observed in different arrangement against a black background, and viewed at different incident angles of the light, which was narrowly concentrated in one direction at a slight angle to the wings surface.

According to Shevtsova and Hansson (2011) the observation and documentation of WIPs do not require a special light source and can be done on dry specimen with intact wings arranged against a black background. In present studies material was illuminated by fiber optic illuminator Cold Light L-150A (Quartz halogen light source color temperature-3200K).

## Results

The present study confirmed that WIPs are present on the dry, minute and semitransparent wings of male scale insects. SEM observations (Fig. 2, 3) showed that membrane of the scale insect wing is characterized by the presence of corrugations and microtrichia.

In case of Hymenoptera and Diptera such membrane corrugations and hair placement together with uneven membrane thickness form stable and taxon specific colour patterns.

Convex ridges of a corrugated wing membrane act as a dioptres to stabilize the interference reflection and eliminate the iridescence effect over a large range of light incidences, it means that provide optical stabilization to WIPs. According to Shevtsova (2012) the convex ridges of a corrugated wing membrane reflect thin film interference colours from the top. When such a wing is tilted these “tops” move along the ridge and show the same interference colour. That is because they keep nearly horizontal at each point and maintain the same distance for the light beams that travel inside the wing.

**Figures 6–13.**
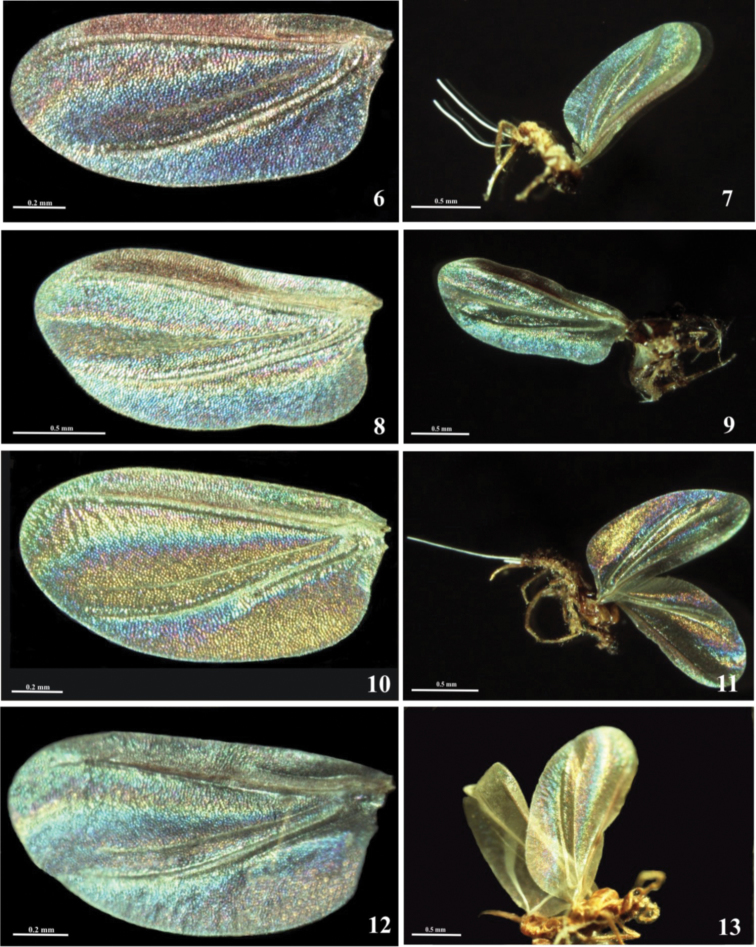
Males with “horizontally striped patterns” of WIPs, subfamilies Eulacaninae and Coccinae: **6–7** WIP of *Sphaerolecanium prunastri* (Boyer de Fonscolombe) **8–9** WIP of *Eulecanium tiliae* (Linnaeus) **10, 11** WIP of *Pulvinaria vitis* **12–13** WIP of *Parthenolecanium corni* (Bouché).

Among examined species all of them exhibited its own WIP which could be distinguished from that of other species. Among the species two main types of patterns could be distinguished: “horizontally striped pattern”, encountered on broad wings of species which Giliomee (1967) assigned to the “Coccus group” (*Pulvinaria*, *Parthenolecanium*) and “Eulecanium group” (*Eulecanium*, *Parthenolecanium* and *Sphaerolecanium*). According to Hodgson’s classification (1994), *Pulvinaria* and *Parthenolecanium* belong to subfamily Coccinae, *Eulecanium* and *Sphaerolecanium* to subfamily Eulecaninae. This pattern here is characterized by the presence of longitudinal stripes of different interference colours on the field between the subcostal and cubital ridges (terminology after Koteja 2008). This striped band is curved a little and the stripes rich the margin of the wing. The main differences between the patterns of this group are connected with the semitriangular region delimited by lower margin of cubital ridge, posterior wing margin up to the wing base and the part which surrounds the first light line. In *Sphaerolecanium prunastri* (Figs 6, 7), this semitriangular region is a deep blue, the region anterior to the cubital ridge rich purple and than blue. In *Eulecanium tiliae* (Figs 8, 9), the main part of mentioned above semitriangular region is blue, but the area which adjoin to the cubital ridge has yellowish and purple stripes.

In *Pulvinaria vitis* (Figs 10, 11), the main part of the region delimited by cubital ridge, posterior wing margin and the margin near the wing base is golden-hued. The same golden color surrounds the first light line. To these golden-hued areas adjoin from above purple-blue stripes. The WIP of the *Parthenolecanium corni* (Figs 12, 13) is quite similar to that of *Pulvinaria vitis* but, in the former species, there is broad blue-purple band on the part which lies below the cubital ridge (in *Pulvinaria vitis* there is only narrow purple-blue stripe). Only narrow yellowish stripes surround the first light line (in *Pulvinaria vitis* there is broad golden band). But the purple-blue band which adjoins to golden-hued area (which surrounds the first light line in *Pulvinaria vitis*) here, in *Parthenolecanium corni* is much broader.

The pattern of the second type of WIP can be described as “ elliptical” - colours follow one by one, and do not form distinct stripes beneath the subcostal ridge which reach the apical margin of the wing, as in previous type.

**Figures 14–19.**
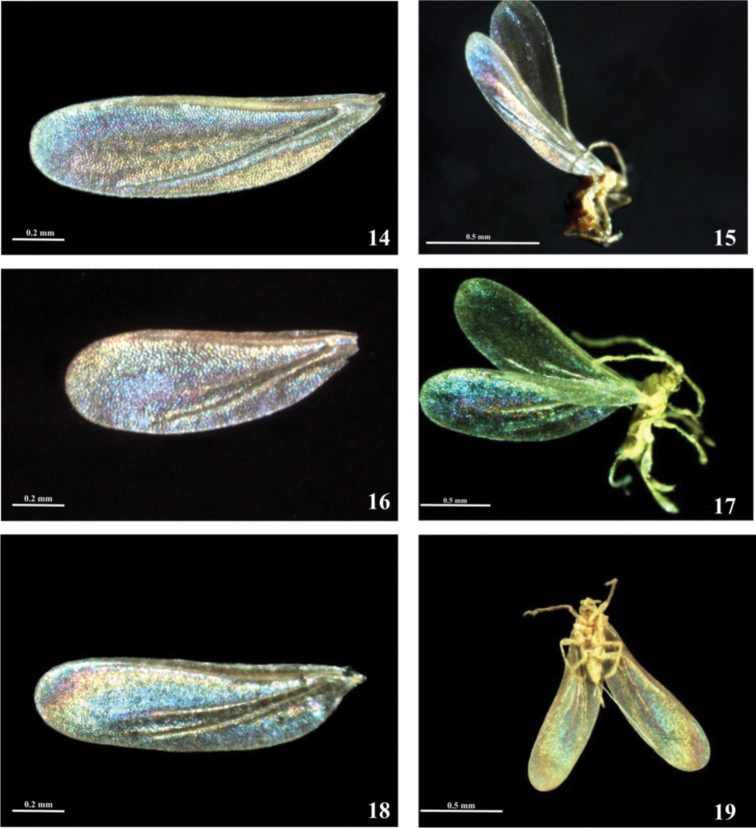
Males with “eliptical” patterns of WIPs, subfamily Eriopeltinae: **14, 15** WIP of *Luzulaspis frontalis* Green **16–17** WIP of *Luzulaspis nemorosa* Koteja. **18–19** WIP of *Eriopeltis lichtensteini* Signoret.

This type of WIP is present on long and narrow wings of *Eriopeltis lichtensteini*, *Luzulaspis frontalis* and *Luzulaspis nemorosa* . The apical part of the wing is covered only by one colour. These two genera were grouped by Giliomee in the “Eriopeltis group” and in the subfamily Eriopeltinae by Hodgson (1994). Likewise, as in species of the previous group (horizontally striped pattern), the WIPs are taxonomically specific, not only at generic level but at the species level too. The patterns of *Luzulaspis frontalis* and *Luzulaspis nemorosa* are significantly different. In *Luzulaspis frontalis* (Figs 14, 15), the area which surrounds the first light line is golden-hued, than from above it turns into purple, than in deep blue and than light blue. Whole part of the membrane which lies beneath the cubital ridge is golden-hued.

In *Luzulaspis nemoralis* inner part, which surrounds the first light line is purple, than colour turns into blue and near the anterior edge of the wing is yellowish-green (Figs 16, 17). All part below the cubital ridge is purple-bluish. In *Eriopeltis lichtensteini* (Figs 18, 19) first light line and cubital ridge are surrounded by blue area. Near the subcostal ridge blue turns into yellowish green. Very characteristic is purple spot near the apical part of the wing.

The WIPs of each of these seven species are significantly different and could provide additional insights into species recognition. These colour patterns are stable and can be seen not only on horizontally arranged wings but also on hole specimen (Figs 7, 9, 11, 13, 15, 17, 19), the only requirements are black background and proper illumination.

## Discussion

The use of wing interference patterns (WIPs) as a morphological character is so new that very little is known about their significance, either to the behavior of the species or in terms of morphological taxonomy, although they have already proven to be useful for generic- and even species–level classifications, particularly in Hymenoptera (Shevtsova and Hansson 2011, Hansson and Shevtsova 2012). These patterns have been even investigated from an evolutionary standpoint for the Cynipoidea (Hymenoptera) (Buffington and Sandler 2011). The preliminary studies outlined above show that each of the seven species has its own WIP, probably after further research, which include more species, these colour patterns could find practical applications in morphological studies of this family.

In other insect groups, such as Diptera and Hymenoptera, it is believed that WIPs are not only a byproduct of physical traits, but probably also function in intra- and interspecific signaling. Thus wing display play a central role in visual courtship communication in several groups of Diptera (e.g. Sepsidae, Tephritidae) and Hymenoptera (e.g. Chalcidoidea: Pteromalidae) (Shevtsova et al. 2011, Shevtsova 2012).

At the present time, in geological history only male Coccoidea possess wings. Females are often sedentary, only have simple unicorneal eyes and all are completely apterous. It seems, therefore, unlikely that these females are able to see sophisticated colour patterns on male’s wings. However Koteja (1990) suspected that “the coccid male at some time had functional mouthparts and well-developed metathoracic wings, and that the female was alate and possesed compound eyes” Shcherbakov (2007) considered that the family Naibiidae is ancestral to the true scale insects. Representatives of the Naibiids were not sexually dimorphic, bouth sexes were presumably feeding and flying. Obviously we cannot check whether WIPs were present on the four-winged ancestors of scale insects, but it is possible that in Mesozoic times, WIPs played a significant role in inter- and intra-specific signaling.

Interestingly, Koteja (2001) noted that male *Porphyrophora* sp. (Margarodidae) performed flight stretching and repositioning of the wings under laboratory conditions. In the same paper, Koteja quoted very similar observations by Jakubski (1929) (“the males made an impression of being ill - they exposed and reposed the wings in hopeless efforts of flight”). Furthermore, both authors noted that the halters were vigorously vibrated when the fore wings were still. The same was observed by Pflugfelder (1939 in Koteja 2001) in males of *Kerria lacca* (Kerridae) when females were nearby. Maybe these examples of wing movement and haltere trembling are remnants of a visual communication by their ancestors, connected with displaying the wings and their patterns.

The types of WIPs seen in this study appear to reflect the affinity between the members of Coccidae. Indeed, the types of WIPs found here might be connected with their ecology, in so far as the “horizontally striped pattern” occurs in species which are present on woody plants: *Sphaerolecanium prunastri* feeds mainly on representatives of Rosaceae, and *Eulecanium tiliae*, *Parthenolecanium corni* and *Pulvinaria vitis* are common poliphagous species also encountered on different woody host plants (Kosztarab and Kozar 1988), while all species with the “elliptical pattern” (*Eriopeltis lichtensteini*, *Luzulaspis frontalis* and *Luzulaspis nemorosa*) feed on monocotyledonous plants: Cyperaceae, Juncaceae and Poaceae (Kosztarab and Kozar 1988). Because of the limited number of observed species this ecological dependance of the pattern might be only a convergence of circumstances but it is worth to checked in other species of this family.

According to Shevtsova et al. (2011) and Shevtsova and Hansson (2011), WIPs are intraspecifically variable and phenotypically plastic in small wasps and diptera, although they are largely uniform among conspecifics and often appear to be characteristic of a species.

On the basis of the examined material, the WIPs of male Coccids appear to be uniform among conspecifics. However, more material from different localities and from different host plants, etc need to be studied to know if there is any intraspecific variability or phenotypical plasticity. If WIPs are used by their bearers for visual communication and, if this signalling system is involved in reproductive isolation and species recognition (Shevtsova 2012), it will be interesting to check the variability between scale insect species belonging to a single genus but which are either allopatric or sympatric, as investigated Hansson and Shevtsova in the eulophid genus *Omphale* Haliday (Shevtsova 2012).

The importance of the adult male structure for the proper understanding of the relationships within the Coccoidea was recognized by Hodgson and Henderson (2004), who considered that no satisfactory system of scale insect classification would be achieved without an understanding of male structure. Most species of scale insects reproduce sexually, but there is a lot of species which are parthenogenetic and reproduce without males, only in some *Icerya* ssp. hermaphroditism has been reported (Gullan and Kosztarab 1997, Gavrilov and Kuznetsova 2007). In case of many others species males are not known at all. Because scale insects are known as serious plant pests in several cases of important, sometimes quarantine species male pheromone traps are used for survey and monitoring. In this case for correct species identification molecular techniques are available (Tobias et al. 2010) and WIPs might support and faciliate species determination.

If the usage of WIPs in coccidology becomes as useful as in the studies on Hymenoptera and Diptera, it will be very important to keep the wings under dry conditions. At present, the most common method of specimen storage prior to making microscopic slides is to preserve the specimens in ethanol. Unfortunately, the WIP colour patterns are invisible on specimens mounted in Canada balsam, while scale insect wings which have been preserved in alcohol either do not show WIPs at all or the patterns are not as bright and clear as those using dry wings.

For a long time, wings of scale insects have been regarded as being poor in morphological features because of their reduced venation and lack of pigments. WIPs might be an additional trait for facilitating species identification. Because studies on scale insect wings are easy and cheap, studies of the wing interference patterns might be a helpful tool in species recognition and for clarifying many taxonomic problems, together with very important and common molecular techniques (Gullan et al. 2010, Tobias et al. 2010, Malausa et al. 2011).

## Conclusions

These preliminary studies confirmed the presence of stable and taxonomically specific wing intereference patterns (WIPs) on scale insects wings. The thin wings of males display vivid structural colour patterns due to thin film interference when viewed against black background using white light. The use of WIPs as a species character in the species studied here has produced some convincing patterns and therefore these small, semitransparent organs should not be regarded as unimportant features anymore. However, if WIPs on male wings are to be studied, it is imperative that are stored dry and not preserved in ethanol or mounted in Canada balsam.

It is intended that these studies will continue, using a wide range of species from different scale insect families and also to consider any effects of intraspecific variation and phenotypic plasticity.
